# Physical Activity Levels During Physical Education Classes and Their Impact on Physical Fitness in 10-Year-Old School Children: A Comparative Study

**DOI:** 10.3390/jfmk9040220

**Published:** 2024-11-04

**Authors:** Vladan Pelemiš, Slobodan Pavlović, Nebojša Mitrović, Ivko Nikolić, Dalibor Stević, Nebojša Trajković

**Affiliations:** 1 Faculty of Education, University of Belgrade, 11000 Belgrade, Serbia; ivko.nikolic@uf.bg.ac.rs; 2 Faculty of Education, University of Kragujevac, 31000 Užice, Serbia; slobodan.b.pavlovic@gmail.com; 3 Faculty of Education, University of East Sarajevo, 76300 Bijeljina, Bosnia and Herzegovina; nebojsa.mitrovic@pfb.ues.rs.ba (N.M.); dalibor.stevic@pfb.ues.rs.ba (D.S.); 4 Faculty of Sport and Physical Education, University of Nis, 18000 Nis, Serbia

**Keywords:** movement, motor skills, primary education, influence

## Abstract

**Background/Objectives**: The aim of this research was to determine the differences in physical fitness according to the level of physical activity (PA) during physical education classes in 10-year-old school children. **Methods**: The research included 315 primary school children (age 10 ± 1.3 years), divided into three groups by level of PA: low, moderate and high. A Eurofit test battery was used to evaluate the physical fitness of children. Physical activity (volume (number of steps) and intensity) was measured using a Coach Gear pedometer and a Suunto Memory Belt heart rate monitor. **Results**: Presented results indicate that there are significant differences between groups of children of both genders in relation to the level of PA. Group of boys with low PA showed lower values in sit and reach (*p* = 0.01), standing long jump (*p* = 0.02), bent arm hang (*p* = 0.04) and polygon backwards (*p* = 0.01) compared to the remaining two groups. Girls with low physical activity showed significant differences in sit and reach (*p* = 0.01) and bent arm hang (*p* = 0.01) compared to the other two groups, while in hand tapping, the high PA group showed better results compared to the other two groups (*p* = 0.03). **Conclusions**: The results reported in this research support the significant effects of PA level on physical fitness in school children. Less active children generally showed poorer physical fitness in both genders.

## 1. Introduction

The quality of physical education (PE) teaching, as foreseen by the curriculum, is met with numerous problems and difficulties in its realization on a daily basis. On the one hand, problems arise from the lack of material resources for realization of PE, which primarily refer to inadequate space for classes, lack of equipment and teaching requisites, and on the other hand, problems are closely related to the quality and structure of classes [1]. An important prerequisite for PE to truly facilitate healthy growing up and prepare children for an active lifestyle is their full engagement and activity in PE classes.

Given the fact that physical activity (PA) is a key feature of PE classes, through PA and in its context, children learn motor skills, learn about PA, get to know themselves and their abilities, communicate with other children in school and develop social skills. Participating in PE classes helps students enhance their physical fitness, and the PA they engage in during these classes contributes to meeting the minimum recommended daily PA level which is at least 60 min each day [2]. Pupils’ achievements in PE classes depend on a number of factors, with the time spent exercising and physically active as one of the most important ones. Pupils effectively acquire motor competence, improve physical fitness, gain positive exercise experiences and build essential confidence only through active participation in class [3]. Consequently, it is necessary for pupils to be adequately engaged, and that primarily refers to the extent and intensity of their activity [4]. The recommended level of moderate-to-vigorous PA in PE classes is 50–80% of teaching time, i.e., the total duration of the class [5]. According to van Sluijs et al., PA is essential in childhood due to its profound effects on both immediate and long-term health outcomes. Authors stated that engaging in regular PA supports not only the development of fundamental motor skills, cardiorespiratory fitness and muscular strength, but also has an impact on lowering the risk of chronic diseases such as cardiovascular disease, obesity and type 2 diabetes, affects mental health, enhances self-esteem and improves cognitive function [6]. 

Physical fitness in children is strongly correlated with their level of PA, with evidence suggesting that more active children exhibit enhanced fitness across multiple domains, including cardiovascular health, strength and flexibility [7]. Engaging in regular, moderate-to-vigorous physical activity is associated with improvements in aerobic capacity and muscular fitness, which are essential for growth, motor development and long-term health [8]. Studies utilizing objective measurements, such as pedometers and heart rate monitors, indicate that children with higher daily step counts and increased intensity levels display superior physical fitness outcomes [6]. Furthermore, PA in childhood is linked to a reduced risk of developing obesity and metabolic disorders, as well as promoting positive psychological benefits, such as improved self-esteem and mental resilience [9].

Although the relationship between PA and physical fitness in children has been well documented, there are still several gaps in understanding how PA levels during PE classes specifically contribute to fitness outcomes. Much of the existing research focuses on overall daily or weekly activity, neglecting the unique role that structured PE classes play in children’s physical development. Additionally, the variability in PE curricula and the way PA is implemented across different schools, school equipment and regions is often overlooked, making it difficult to generalize findings. Furthermore, while some studies touch on gender differences, the specific ways boys and girls may respond differently to the same levels of PA during PE remain underexplored [10]. Another critical gap lies in the reliance on self-reported data or general fitness assessments, with few studies utilizing objective tools, such as pedometers or heart rate monitors, to measure PA during PE. One of the structural problems affecting the functioning of PE in primary schools can be alleviated by determining the level of PA for boys and girls [11]. In order to achieve the full effectiveness of PE, the intensity and volume of physical activity of students must be determined at that level, at which they will leave traces of adaptation to new higher loads. While prior studies have explored PA during school hours or in leisure time, limited attention has been given to examining the specific contribution of PE classes to meeting recommended PA levels and improving fitness outcomes in children. Our study addresses this gap by providing a comparative analysis of physical fitness based on varying activity levels during PE classes. This research should answer questions about what happens to the physical fitness of school-aged children after assessing their level of PA in PE classes. The findings could influence new curriculum planning in PE in schools, which would have a better effect on the anthropological status of children. Therefore, the aim of this research was to determine the differences in physical fitness of children in relation to the level of their PA in their PE classes.

## 2. Materials and Methods

### 2.1. Participants 

The research included 315 school children from primary school (ages 10 ± 1.3 years), of which 177 were boys and 138 were girls. Participants were selected by random sampling from the territory of western Serbia. They were divided into three groups based on the level of PA determined in PE classes (low; moderate; or high). All children regularly attended PE classes in schools three times a week, with each class lasting 45 min. Children were excluded from the study if the pedometer or heart rate monitor failed to provide accurate or complete data due to improper use or there were missing measurements. Additionally, any child who did not fully participate in or complete the PE class (e.g., due to early departure, illness, injury or refusal to continue) was excluded from the analysis to maintain the reliability of the results. Classes were held according to the prescribed curriculum. School authorities and parents were informed of the purpose and content of the research and their written consent was ensured, in accordance with the Convention on the Rights of the Child. Parents of children were provided with a survey questionnaire prior to the realization of the research, outlining the plan and course of the study, and they authorized research on their children with their signatures according to the Declaration of Helsinki. The research was approved according to the decision of the Ministry of Education, Sciences and Technological Development of the Republic of Serbia (number 149-32/2022), which includes testing of physical fitness, anthropometric measurement and surveying of primary school children.

### 2.2. Procedures 

The research was conducted in the 2022/2023 school year, during the months of March, April and May, as part of regular PE classes in five elementary schools in western Serbia. The measurement of body composition parameters was firstly carried out, followed by physical fitness testing. After that, PA of the children during PE classes was measured. Teaching units were largely based on sports games. During all students’ activities in PE classes, care was taken to minimize the teachers’ direct influence on the level of engagement, that is, on the PA of the students. Accordingly, appropriate methods and forms of class work were selected. Emphasis was placed on movement tasks that allow students to independently determine how active they will be in class. 

Basic anthropometric measures were selected as a sample of measuring instruments: (1) body height (cm) was measured using a Martin anthropometer and (2) body weight (0.1 kg) was measured by the InBody 230 (Biospace Co., Ltd., Seoul, Republic of Korea).

Body composition was assessed using (1) total muscle mass (0.1 kg), measured by the InBody 230 (Biospace Co., Ltd., Seoul, Republic of Korea) and (2) total body fat (0.1 kg), measured by the InBody 230 (Biospace Co., Ltd., Seoul, Republic of Korea).

BIA analysis is a rapid, non-invasive method for assessing the body composition, in field and clinical settings. It has been used in previous research studies on a similar sample of participants [12] and has proven to be valid and reliable. The authors also state that it has become a reference method in research studies of body composition analysis and that in comparison with DEXA, InBody (Biospace Co., Ltd., Seoul, Republic of Korea) has been shown to produce highly accurate (r = 0.974) results. 

The standardized physical fitness test battery Eurofit, with good metric characteristics, was applied in the research [13]. The following tests were applied: for body coordination—Polygon backwards (0.1 s); for explosive strength—standing long jump test (cm); for speed—running 20 m (0.1 s); for arm and shoulder strength—bent arm hang (0.1 s); for movement frequency—hand tapping (freq.); for flexibility—sit and reach (0.1 cm); and for repetitive core strength—sit ups (freq.). 

Physical activity (volume (number of steps) and intensity) of students in PE classes was measured using a CoachGear pedometer and a Suunto Memory Belt heart rate monitor [3]. This device is a newer-generation device and is a reliable heart rate monitor. It is easy to use and does not require lengthy training for application. Before use, the following values are entered into the device: age, body mass and body height. The device is then attached to the chest area with an electrode in the middle. The beep confirms that the unit is operational. When the measurement is complete, the device data are read by a reader and directly transmitted to the computer. The device has a graphic record of pulse values during PA as well as time spent in different intensity zones. Intensity zones are set in such a way that three categories (zones) of PA are defined: low-intensity zone (light physical activity, abbr. LPA = resting heart rate + x < 25% of resting heart rate value); moderate-intensity zone (moderate physical activity, abbr. MPA = resting heart rate + 25% + x < 50% of resting heart rate value); and high-intensity zone (vigorous physical activity, abbr. VPA = resting heart rate + x > 50% of resting heart rate value). The boys took an average of 2321 steps and engaged in vigorous physical activity (VPA) for a total of 22.05 min. On average, they spent the same amount of time completing low- (LPA) and moderate-intensity (MPA) activity (11 min each). On average, the girls were engaged in high-intensity physical activity (VPA) for about 21 min, that is, 12 min each in low- and moderate-intensity physical activity (LPA, MPA). As a representative indicator of intensity of a pupil’s PA in class, total time spent by the student in the zone of vigorous physical activity (VPA) was used for further analyses. By placing the device in the middle of the chest and putting it into operation, the resting heart rate was recorded. These values determined exercise intensity zones. Each intensity zone was related to a certain degree of exertion, which pupils achieved during PE activities. The upper limit of the low-intensity zone (light physical activity—LPA) was obtained when the resting heart rate value was summarized with a value that is less than 25% of the resting heart rate. Further, the moderate-intensity zone (moderate physical activity—MPA) was obtained by summarizing the resting heart rate values and heart rate values in the range of 25–50% of the resting heart rate, whereas the zone of intense physical exercise was obtained by summarizing the resting heart rate values and values greater than 50% of the resting heart rate. Children were divided into the three zones according to the method of Pate et al. [14]. 

### 2.3. Statistical Analysis

Statistical analyses were performed using the SPSS software version 23 (v21.0., SPSS Inc., Chicago, IL, USA). Since the variables of volume and intensity of students’ PA, which were estimated in PE classes, come from different metric spaces, their standardization was necessary. Pupils’ groups were determined using the squared Euclidean distance index of both variables mentioned above with Ward’s hierarchical cluster analysis method. Basic descriptive statistics were established for all variables and mean and standard deviation (SD) were used for analysis and comparison. Given the fact that it was necessary to examine group differences at the level of the entire sample of boys and girls in the tested variables (determining differences in physical fitness and body composition), a multivariate analysis of variance (MANOVA) was used, and individual statistically significant differences were determined by univariate analysis of variance (ANOVA).

## 3. Results

Table 1 and Table 2 give an overview of the division of groups using Ward’s hierarchical clustering procedure of grouping participants. Differences in physical fitness and body composition between the gender-formed groups were determined using multivariate analysis of variance and individually for each variable using univariate analysis of variance. The results presented in Table 1 and Table 2 indicate that there are differences between the groups of male and female respondents, so both tables are summarized and presented in as short a form as possible. 

The group of boys with low PA (values shown in Table 1) showed the lowest average values of physical fitness compared to the remaining two groups. Specifically, the group of boys with low PA showed lower values in sit and reach (*p* = 0.01), standing long jump (*p* = 0.02), bent arm hang (*p* = 0.04) and polygon backwards (*p* = 0.01) compared to the remaining two groups. It can be seen from Table 1 that the group with high PA showed better results in physical fitness in comparison with groups with low and moderate PA. 

The group of girls with high PA showed the highest average values for physical fitness in all measured variables, while the group with low PA showed the lowest scores compared to the other groups tested (Table 2). Specifically, girls with low PA showed significant differences in sit and reach (*p* = 0.01) and bent arm hang (*p* = 0.01) compared to the other two groups, while in hand tapping, the high PA group showed better results compared to the other two groups (*p* = 0.03).

## 4. Discussion

The aim of the current research was to analyze the differences in physical fitness of 10-year-old school children in relation to the level of their PA in PE classes. The results presented indicate that there are differences between the groups of children in both genders in relation to the level of PA. Moreover, differences were more pronounced in boys than girls as indicated by separate analyses conducted for each gender. This difference could be explained to some extent at this age by the greater influence and amount of PA that boys exhibit and by the variety that is present in their extracurricular activities, and in most cases, it is carried out in the forms of polystructural and complex physical activities. Girls’ PA in extracurricular activities is somewhat different for their needs, and in most cases at this age, it is based on esthetic sport activities, as confirmed by the findings [15]. Further, boys tend to engage in higher-intensity physical activities and sports outside of school more frequently, which further enhances their fitness levels. Additionally, boys are often encouraged to participate in competitive and physically demanding activities, whereas girls may engage in more moderate-intensity activities, which could influence fitness outcomes. 

Regarding the body weight status of boys and girls, body weight did not represent a significant predictor in which differences were observed between groups of boys according to the level of PA. Opposite findings were reported in girls, where these differences exist for body weight. Research shows that boys tend to be more physically active than girls, which may account for differences in body weight and composition between genders [16]. Studies have demonstrated that boys generally engage in higher levels of moderate-to-vigorous physical activity, both in and outside of school, compared to girls [17,18]. This increased activity in boys is likely a contributing factor to their lower body fat percentages and greater muscle mass, as regular PA promotes healthier body composition by increasing energy expenditure and promoting muscle development. In contrast, girls’ lower PA levels may contribute to higher body fat accumulation, resulting in differences in body weight status between the sexes. 

As already mentioned, PA in class was measured by distance covered (number of steps) and intensity (time spent in the intensive physical activity zone). Viewed through body composition (fat and muscle component), differences were observed in boys with respect to the level of PA, which is in line with previous findings [3]. Such an indicator is not surprising, since in relation to each other, a higher percentage of muscle mass and a smaller fat component is a prerequisite for greater general physical activity and, consequently, PA in PE classes. Less fatty tissue and a more muscular type of build, free of undesirable excess body weight, would allow an individual to potentially be more capable of greater PA, as confirmed by this research. In girls, differences are defined only in the fat component, while in the muscular component, they are not. However, in girls, when differences in body weight are added, it is concluded that body voluminosity is a characteristic that significantly differentiates the level of PA during PE, which has been confirmed in some studies [19,20]. The fact that boys do not differ in body weight in relation to the level of PA in PE classes is in some ways an unusual occurrence. It can be said that boys are generally more motivated, in the broadest sense [21], to perform physical activities and activities in PE classes, and therefore body weight did not represent a characteristic by which boys would differ. The observed average values of muscle and fat components in boys in which they differ from one another indicate more intense processes of growth and development in this age period. The distribution of fatty tissue at this age depends on hormones and shows the role of gender. There is relatively more fat in girls than in boys, so by examining the arithmetic means of the groups between the genders, a higher level of total fat in girls can be observed. This is due to the early entry of girls into the phase of intense growth and development, which at this age is manifested by slightly higher values of total fat in the body, and soon there will be a phase of longitudinal dimensionality and bone growth in length in girls. This is justified by the fact that the cells before the growth phase are first filled with fat as a type of fuel that they will use to expand bone growth in length [22]. 

Understanding the impact of PA levels on children’s physical fitness is essential for developing targeted interventions aimed at promoting health through active lifestyles. Schools, families and communities play an integral role in fostering environments that support regular PA for children, particularly given the increasing prevalence of sedentary behaviors driven by technology use. Children in the current study showed differences in physical fitness according to the level of PA in PE class. Specifically, boys showed differences in coordination, flexibility, explosive and static strength, while girls differed significantly in movement speed, flexibility and static strength. It is interesting to note that differences in both genders are most pronounced in flexibility. The least active children had the lowest flexibility scores, which is in line with some previous research [20]. More active children are more flexible due to their movement experience, because with adequate movement amplitude, they are more likely to achieve higher levels of PA in the PE class [23]. Regarding the bent arm hang test and the strength of the arms and shoulder, there is also a difference in both genders. The group which was the least active in PE classes achieved the lowest average values on the test. Perhaps the reasons for these results are supported by the fact that less active children had a higher percentage of fat component and girls also had greater body weight, which led to poorer results on the arm and shoulder strength test [24]. Differences in coordination were manifested in boys, while in girls, these differences were not observed. It is noticeable that boys from the group that was the least active had the poorest results in the coordination test, which suggests that coordination is a significant factor in manifesting differences in the level of PA of pupils in PE class [25]. 

The reason for this may be found in the fact that more physically active boys have a richer movement memory, which enables them to cope better with newly acquired movement tasks [26]. Some children have an “active phenotype”, while other children are less physically active, regardless of their environment. Children may differ according to the internal mechanisms of biological control of PA [27]. It should also be noted that the findings to date indicate that persons with a higher percentage of fat component and reduced PA are lagging behind in coordination with the population of normally nourished people [28]. According to the author, the reasons for these findings are because overweight individuals have a problem with the integration and processing of sensory information, which results from impairment in the functioning of perceptual gestalt motor functions. What is emerging as a general view in most research is that if one assesses coordination that requires engagement of larger body segments or movement of the whole body, overweight individuals achieve poorer results [29,30]. Differences in the segmental speed of movement were observed in girls, while they were absent in boys. Girls from the least active group had, on average, lower scores than the other two groups, which suggests that the level of PA influences the expression of differences in measuring the speed of individual movements in girls, which is in agreement with some studies [31]. When it comes to explosive strength of the lower extremities, differences were observed in boys but not in girls. Also, it should be noted that boys from the group of least active students had the worst results in the expression of explosive strength of the lower extremities. This fact leads to the conclusion that more active boys in groups completing moderate- and high-intensity exercise possessed greater leg strength, which further means that greater engagement in the class can stimulate greater lower extremity strength [32]. These results are not surprising given the fact that in this study, PA was measured by engagement of the lower extremities. Due to the increased PA of the most active group, compared to the other two groups of children, development of lower extremity strength probably occurred. Pupils from the most active group, through various forms of movement in PE classes, primarily realizing higher intensity of PE, initiated the development of lower extremity strength, where differences were also noted. 

A potential limitation of this study is that it focuses solely on PE during PE classes, which may not fully capture the overall PE levels of children throughout the day, such as during free play or extracurricular activities. The cross-sectional design limits the ability to establish causal relationships between PE and physical fitness, and the lack of longitudinal data prevents the assessment of long-term effects. Another limitation is the potential influence of unmeasured variables, such as socioeconomic status, nutrition or home environment, which could impact both PE levels and fitness outcomes. Finally, the study sample may not be fully representative of all school children, limiting the generalizability of the findings. However, the study has its strengths and practical implications. Observations made in this research highlight the potential importance of encouraging PE during PE classes, on the basis that differences in physical fitness are manifested in younger school-aged children, in order to achieve more efficient organization and management of PE classes. Proper application of the PE level, primarily by dosing, can significantly affect biological growth and development of children. 

## 5. Conclusions

The results reported in this research support the significant effects of PA level on physical fitness in school children. Less active children generally showed poorer physical fitness in both genders. Based on the above facts, it is necessary to carry out a certain analysis of the realization of PE classes and to plan them adequately. The results highlight the importance of regular PE in improving children’s physical fitness. Additionally, our findings provide teachers with specific insights into both the quantity (e.g., optimal frequency and duration) and quality (e.g., diversity and structure) of physical activities suitable for this age group. Accordingly, PE teachers need to effectively promote PE in education classes, aiming to support children’s healthy development and prepare them for an active lifestyle.

## Figures and Tables

**Table 1:** Descriptive statistics and differences between groups in boys.

	Low PA		Moderate PA		High PA			
Variable	(*n* = 45)		(*n* = 65)		(*n* = 67)		f	*p*
	M	SD	M	SD	M	SD		
Body height (cm)	144.52	11.32	143.53	6.78	149.64	9.21	0.74	0.39
Body weight (kg)	39.22	8.11	37.38	8.06	38.21	8.67	0.02	0.89
Muscle component (%)	13.89	3.23	15.17	2.68	16.22	3.13	1.75	0.01
Fat component (%)	9.39	6.13	8.34	4.69	8.01	3.91	8.05	0.01
Running 20 m (0.1 s)	4.91	0.66	4.33	0.42	4.19	0.25	2.76	0.10
Polygon backwards (0.1 s)	25.88	9.44	23.67	8.08	22.55	7.69	8.08	0.01
Hand tapping (freq.)	37.03	10.10	39.07	9.09	41.15	9.65	2.99	0.09
Seated forward bend (cm)	45.54	9.37	50.50	9.30	52.03	8.77	9.69	0.01
Standing long jump (cm)	134.11	19.87	140.96	20.55	145.09	17.49	13.35	0.02
Bent arm hang (0.1 s)	8.87	7.17	11.20	9.84	13.08	8.88	2.52	0.04
Sit ups (freq.)	27.44	6.55	29.78	7.19	31.25	6.79	1.27	0.10
							F = 6.98	P = 0.01

Legend: M—arithmetic mean, SD—standard deviation, f—univariate F test; *p*—level of statistical significance of univariate F test; F—multivariate Wilks’ F test; P—statistical significance of multivariate F test.

**Table 2:** Descriptive statistics and differences between groups in girls.

	Low PA		Moderate PA		High PA			
Variable	(*n* = 47)		(*n* = 59)		(*n* = 32)		f	*p*
	M	SD	M	SD	M	SD		
Body height	140.69	6.66	142.79	7.44	142.64	9.12	1.12	0.29
Body weight	39.56	8.91	36.56	7.84	37.21	8.76	0.28	0.03
Muscle component	12.16	3.78	14.26	4.69	14.22	3.31	3.11	0.08
Fat component	9.71	1.99	9.00	2.62	8.01	3.19	3.99	0.02
Running 20 m	4.89	0.45	4.49	0.44	4.25	0.24	1.57	0.11
Polygon backwards	27.10	7.11	25.30	8.10	23.45	7.69	1.61	0.27
Hand tapping	36.27	8.33	37.87	8.42	39.15	9.55	6.71	0.03
Seated forward bend	52.84	11.10	54.94	10.58	56.03	8.22	2.59	0.01
Standing long jump	131.31	20.20	134.13	19.11	141.09	16.49	0.45	0.50
Bent arm hang	6.78	9.17	9.28	8.50	11.08	7.16	2.03	0.01
Sit ups	27.36	5.49	28.46	6.59	30.25	4.79	3.05	0.08
							F = 4.59	P = 0.00

Legend: M—arithmetic mean, SD—standard deviation, f—univariate F test; *p*—level of statistical significance of univariate F test; F—multivariate Wilks’ F test; P—statistical significance of multivariate F test.

## Data Availability

The data presented in this study are available on request from the corresponding author.

## References

[1] Hardman K. (2008). Physical education in schools: A global perspective. Kinesiology.

[2] Pelemiš V., Pavlović S., Mandić D., Radaković M., Branković D., Živanović V., Milić Z., Bajrić S. (2024). Differences and Relationship between Body Composition and Motor Coordination in Children Aged 6–7 Years. Sports.

[3] Pavlović S., Pelemiš V., Marković J., Dimitrijević M., Badrić M., Halaši S., Nikolić I., Čokorilo N. (2023). The Role of Motivation and Physical Self-Concept in Accomplishing Physical Activity in Primary School Children. Sports.

[4] Kasabova L. (2019). Some changes in the psychomotorism of students with different levels in motor activity. Trakia J. Sci..

[5] Kim Y., Lochbaum M. (2017). Objectively measured physical activity levels among ethnic minority children attending school-based afterschool programs in a high-poverty neighbourhood. J. Sports Sci. Med..

[6] Van Sluijs E.M., Ekelund U., Crochemore-Silva I., Guthold R., Ha A., Lubans D., Oyeyemi A.L., Ding D., Katzmarzyk P.T. (2021). Physical activity behaviours in adolescence: Current evidence and opportunities for intervention. Lancet.

[7] Tomkinson G.R., Lang J.J., Tremblay M.S. (2019). Temporal trends in the cardiorespiratory fitness of children and adolescents representing 19 high-income and upper middle-income countries between 1981 and 2014. Br. J. Sports Med..

[8] Smith J.J., Eather N., Weaver R.G., Riley N., Beets M.W., Lubans D.R. (2019). Behavioral correlates of muscular fitness in children and adolescents: A systematic review. Sports Med..

[9] Poitras V.J., Gray C.E., Borghese M.M., Carson V., Chaput J.P., Janssen I., Katzmarzyk P.T., Pate R.R., Connor Gorber S., Kho M.E. (2016). Systematic review of the relationships between objectively measured physical activity and health indicators in school-aged children and youth. Appl. Physiol. Nutr. Metab..

[10] Kretschmer L., Salali G.D., Andersen L.B., Hallal P.C., Northstone K., Sardinha L.B., Dyble M., Bann D., International Children’s Accelerometry Database (ICAD) Collaborators (2023). Gender diferences in the distribution of children’s physical activity: Evidence from nine countries. Int. J. Behav. Nutr. Phys. Act..

[11] Prskalo I. (2015). Kinesiology of free time. Croat. J. Educ. Hrvat. Časopis Za Odgoj..

[12] Meredith-Jones K.A., Williams S.M., Taylor R.W. (2015). Bioelectrical impedance as a measure of change in body composition in young children. Pediatr. Obes..

[13] Grgic J. (2023). Test–retest reliability of the EUROFIT test battery: A review. Sport Sci. Health.

[14] Pate R.R., Baranovski T., Dovda M., Trost S.G. (1996). Monitoring the physical activity of young children. Med. Sci. Sports Exerc..

[15] Pelemiš V., Ujsasi D., Srdić V., Džinović D., Pavlović S. (2019). Analysis of the motor status of younger school age children in relation to their nutritional status. Facta Univ. Ser. Phys. Educ. Sport.

[16] Brazo-Sayavera J., Aubert S., Barnes J.D., González S.A., Tremblay M.S. (2021). Gender differences in physical activity and sedentary behavior: Results from over 200,000 Latin-American children and adolescents. PLoS ONE.

[17] Verloigne M., Van Lippevelde W., Maes L., Yıldırım M., Chinapaw M., Manios Y., Androutsos O., Kovács É., Bringolf-Isler B., Brug J. (2012). Levels of physical activity and sedentary time among 10-to 12-year-old boys and girls across 5 European countries using accelerometers: An observational study within the ENERGY-project. Int. J. Behav. Nutr. Phys. Act..

[18] Steene-Johannessen J., Hansen B.H., Dalene K.E., Kolle E., Northstone K., Møller N.C., Grøntved A., Wedderkopp N., Kriemler S., Page A.S. (2020). Variations in accelerometry measured physical activity and sedentary time across Europe-harmonized analyses of 47,497 children and adolescents. Int. J. Behav. Nutr. Phys. Act..

[19] De Rezende L.F., Azeredo C.M., Canella D.S., Claro R.M., de Castro I.R., Levy R.B., Luiz O.D. (2014). Sociodemographic and behavioral factors associated with physical activity in Brazilian adolescents. BMC Public Health.

[20] Aphamis G., Ioannou Y., Giannaki C.D. (2019). Physical fitness and obesity levels during an academic year followed by summer holidays: An issue of insufficient time for physical activity. Int. J. Adolesc. Med. Health.

[21] Slingerland M., Haerens L., Cardon G., Borghouts L. (2014). Differences in perceived competence and physical activity levels during single-gender modified basketball game play in middle school physical education. Eur. Phys. Educ. Rev..

[22] Živanović V., Branković D., Pelemiš V. (2018). Gender Differences in Children Related to the Body Composition and Movement Coordination. Croat. J. Educ. Hrvat. Cas. Odgoj. Obraz..

[23] Faigenbaum A.D., Bush J.A., McLoone R.P., Kreckel M.C., Farrell A., Ratamess N.A., Kang J. (2015). Benefits of strength and skill-based training during primary school physical education. J. Strength Cond. Res..

[24] Gerber B.P., Pienaar A.E., Kruger A., Ellis S. (2014). Interrelations between anthropometric and fitness changes during mid-adolescence in boys: A 2-year longitudinal study. Am. J. Hum. Biol..

[25] Pereira S., Reyes A., Moura-Dos-Santos M.A., Santos C., Gomes T.N., Tani G., Vasconcelos O., Barreira T.V., Katzmarzyk P.T., Maia J. (2020). Why are children different in their moderate-to-vigorous physical activity levels? A multilevel analysis. J. Pediatr..

[26] Lenhart C.M., Hanlon A., Kang Y., Daly B.P., Brown M.D., Patterson F. (2012). Gender disparity in structured physical activity and overall activity level in adolescence: Evaluation of youth risk behavior surveillance data. Int. Sch. Res. Not..

[27] Eisenmann J.C., Wickel E.E. (2009). The biological basis of physical activity in children: Revisited. Pediatr. Exerc. Sci..

[28] Gentier I., D’Hondt E., Shultz S., Deforche B., Augustijn M., Hoorne S., Verlaecke K., De Bourdeaudhuij I., Lenoir M. (2013). Fine and gross motor skills differ between healthy-weight and obese children. Res. Dev. Disabil..

[29] Savelsbergh G.J., Bennett S.J., Angelakopoulos G.T., Davids K. (2005). Perceptual-motor organization of children’s catching behaviour under different postural constraints. Neurosci. Lett..

[30] D’Hondt E., Deforche B., Gentier I., De Bourdeaudhuij I., Vaeyens R., Philippaerts R., Lenoir M. (2013). A longitudinal analysis of gross motor coordination in overweight and obese children versus normal-weight peers. Int. J. Obes..

[31] Milić M., Grgantov Z., Katić R. (2012). Biomotor status and kinesiological education of girls aged 10 to 12 years–example: Volleyball. Coll. Antropol..

[32] Lirgg C.D., Gorman D.R., Merrie M.D., Hadadi A.A. (2018). Effect of a bicycling unit on the fitness of middle school students. Phys. Educ..

